# Lifestyle modification and hypertension: prescription patterns of Nigerian general practitioners

**DOI:** 10.11604/pamj.2020.35.130.19278

**Published:** 2020-04-20

**Authors:** Olagoke Korede Ale, Rotimi William Braimoh, Adewole Adebiyi, Janet Ngozi Ajuluchukwu

**Affiliations:** 1 Cardiology Unit, Department of Medicine, College of Medicine, University of Lagos, PMB 12003, Lagos, Nigeria; 2 Nephrology Unit, Department of Medicine, College of Medicine, University of Lagos, PMB 12003, Lagos, Nigeria; 3 Cardiology Unit, Department of Medicine, College of Medicine, University of Ibadan, Ibadan, Nigeria

**Keywords:** Lifestyle modification prescription, blood pressure control, hypertension, general medical practitioners, Nigeria

## Abstract

**Introduction:**

Implementation of lifestyle modification (LM), a cornerstone of hypertension control has been reported to be more challenging than pharmacotherapy. We studied the LM prescription patterns of general medical practitioners (GPs) in Lagos, Nigeria for blood pressure control.

**Methods:**

GPs were assessed using anonymous self-administered questionnaire on the prescription of salt restriction, weight management, cessation of tobacco use, physical exercise, and consumption of DASH-like diet for blood pressure control. Chi-square, Fisher's exact and Student t-test were used to test for differences as appropriate. Logistic regression model was constructed to identify the determinants of adequate LM prescription.

**Results:**

A total of 213 GPs (38% females) participated in the survey. LM prescription was over 90% for the following: salt restriction (96.7%), tobacco cessation (94.8%), weight management (94.4%). The remaining were 81.2% and 75.1% for healthy diet and physical activity respectively. The median LM prescription score (of the GPs) was 18.0 [15.0-50.0]. The single significant predictor of adequate LM prescription was total patient load of the GPs (AOR:0.98, 95% CI: 0.97-0.99, p=0.006). Eleven (5.2%), 190 (89.2%), and 12 (5.6%) GPs initiated LM prescription at blood pressure values >140/90mmHg, =140/90mmHg and <140/90mmHg respectively. LM initiation at BP <140/90mmHg was associated with female gender, shorter work experience, working in tertiary care facility and ignorance about hypertension prevention (p<0.05).

**Conclusion:**

LM is widely prescribed for the treatment of hypertension, but rarely prescribed for its prevention in Nigeria. Interventions to reduce physician's patient load may engender improved LM prescription.

## Introduction

Unhealthy lifestyle including unhealthy diet, physical inactivity, tobacco use and harmful use of alcohol may cause blood pressure (BP) elevation as well as initiate other cardiovascular risk factors such as diabetes, dyslipidemia and excessive body weight [1,2]. Civilization breeds unhealthy lifestyle noted in physical inactivity, as well as in transition to more processed foods from staple cereals, vegetables, and fruits [1,2]. All these are drivers of increasing hypertension (HTN) prevalence and poor BP control. The above underpins the assertion by Cooper and Rotimi in 1997 that rural African social environment is one of the kindest to the human cardiovascular system [3]. Over the past decades, growing modernization in Africa has contributed to her being the World Health Organization (WHO) region with the highest prevalence of hypertension [2,4]. Appropriate lifestyle modification (LM) might delay or prevent the onset of HTN in normotensive individuals [5,6]. It might also prevent the initiation of drug therapy in grade 1 hypertension [5,6]. The use of LM might augment BP reduction in hypertensive individuals already on pharmacotherapy, resulting in a reduction in the number and/or doses of antihypertensive drugs [5,6]. Clinical studies have shown that the BP-lowering effect of targeted LM is comparable to single antihypertensive drug therapy [7]. HTN is often co-existent with other cardiovascular (CV) risk factors [5]. These co-existing CV risk factors usually potentiate one another leading to a total CV risk greater than the sum of its individual components [5,6]. Thus, in addition to its BP lowering effect, LM could contribute to the control of other coexisting CV risk factors and clinical conditions [5]. The potential of LM to achieve the above with an additional value of reduced/no exposure to the potential adverse effects of drug therapy makes it a cornerstone of BP control [5]. Since 2013, class IA recommendation status was assigned to LM by the European hypertension guidelines [5]. These lifestyle interventions include weight control, cessation of tobacco use, regular physical exercise, moderation of alcohol intake, salt restriction, and consumption of a Dietary Approaches to Stop Hypertension (DASH)-like diet [1,5,6,8]. It is instructive to note that Kempner diet, which is composed primarily of rice and fruits was one of the earliest methods of hypertension treatment [9]. Kempner diet: a low calorie, protein, fat and sodium diet lowered blood pressure in hypertensive individuals, though it was associated with weight loss and ketosis [9]. It has been documented that despite the clear evidence of its usefulness and benefits, LM remains the most neglected aspect of BP control [10]. World Health Organization has further identified low literacy levels and income as important barriers to lifestyle modification programs in developing countries [11]. Additionally, caregivers contribute to poor HTN control in sub Saharan Africa [12,13]. The contribution of caregivers to the poor utilization of LM measures has been identified as follows: (a) uncertainties regarding when and how to implement LM, (b) provision of inadequate LM information to patients, (c) poor motivation of patients to accept the need for LM, and (e) poor follow up of patients to ensure adherence to LM [14,15]. The aim of this study was to evaluate the LM prescription patterns for BP control by Lagos based general medical practitioners (GPs).

## Methods

To study the prescription of LM, a survey of 213 general practitioners (GPs) based in Lagos was carried out using anonymous self-administered questionnaires. The study group consisted of consecutive consenting GPs attending continuing medical education programs in Lagos. Physicians with post graduate training in internal medicine or any of its sub-specialties were excluded from the survey. Likert-type scale responses were used to evaluate the physicians' prescription of LM for BP control. The LM assessed were salt restriction, weight management, cessation of tobacco use, regular physical exercise, and consumption of a healthy DASH-like diet [5,6,8]. Ethical clearance (reference number: ADM/DCST/HREC/APP/1422) was obtained from the Lagos University Teaching Hospital Health Research Ethical Committee. All statistical data were analyzed using Statistical Package for the Social Sciences (SPSS, version 20.0). Likert-type scale responses were transformed into binary responses of yes (“A= always done” and “B= often or usually done”) and no (“C= sometimes done”, “D= occasionally done”, “E= rarely or never done”) responses. In addition, the Likert-scale responses A, B, C, D, and E were transformed into ordinal scale scores of 4,3,2,1, and 0 respectively. A composite LM prescription score representing the degree of LM prescription was generated from the sum of the ordinal scores. Adequate LM prescription was defined as LM prescription score of ≥18. The GPs were categorized by experience into two groups: ≤10 and >10 years. Descriptive statistics was used to report the findings. Categorical variables were expressed as proportions, while continuous variables were expressed as means± SD. The chi-square or Fisher's exact tests were used as appropriate to test for differences in proportions, while the Student t-test was used to test for the differences in means. In instances of missing responses in the questionnaire items, the number of responses available for each item was reported, and relative frequencies were reported as adjusted percentages. A binary logistic regression model was constructed to identify the determinants of adequate LM prescription (score of ≥18) using the general practitioner's characteristics of gender, practicing experience, practice types and patient load (total and hypertensive) as predictors. All tests were two-sided, and values were considered statistically significant if p < 0.05.

## Results

A total of 213 general practitioners (62% male, n= 132), participated in the survey. The age of the cohort ranged from 27 - 67 years with a mean age of 37.27± 9.31 years. The GPs had been in medical practice for 3 - 38 years with a mean practice experience of 12.21± 9.63 years. One hundred and fifty-five (72.8%) and 58(27.2%) practiced in private and public healthcare facilities respectively. One hundred and forty-two (66.7%) and sixty-four (30%) practiced in primary and secondary healthcare centres respectively. Seven GPs (3.3%) practiced in facilities affiliated to tertiary care centres. The GPs attended to 6-99 patients weekly with an average weekly patient load of 71.23± 27.32. On weekly basis, 1 - 80 hypertensive patients (mean: 20.00± 16.25) were attended to by the GPs. The median LM prescription score of the physicians was 18.0 (15.0 - 20.0). Table 1 summarizes LM prescription of the GPs according to (a) their work experience, (b) the type of health institution of practice, and (c) their knowledge of HTN prevention. General practitioners with >10 years' experience prescribed LM for BP control more than those with ≤10 years' experience. GPs practicing in facilities affiliated to tertiary care facilities prescribed LM for BP control more than those practicing in primary and secondary care facilities. The LM prescription pattern of GPs in public and private practices were similar. One hundred and one (47.4%) GPs knew that HTN is preventable. One hundred and seventy-eight (83.6%) GPs knew that HTN onset can be delayed. All but one respondent (99.5%, n= 212) adjudged LM important for BP control. Knowledge of HTN prevention by the respondents was associated with better LM prescription. However, knowledge that HTN onset can be delayed did not influence LM prescription. The GPs' LM prescription for BP control according to their experience and weekly patient load is shown in Table 2. Table 3 shows the relationship between the physicians' experience, practice type and level, patient load, knowledge about HTN onset delay/prevention and their threshold for prescribing LM. Lifestyle modification was prescribed at BP >140/90mmHg, =140/90mmHg, and <140/90mmHg by 11(5.2%), 190(89.2%) and 12(5.6%) physicians respectively. The threshold for prescribing LM was independent of the type of practice (private vs. government). However, female gender, shorter experience, lighter patient load and affiliation of practice to tertiary care facility were associated with LM initiation at BP <140/90 mmHg. The threshold for prescribing LM was independent of the physicians' knowledge of HTN prevention. Using binary logistic regression, selected physicians' characteristics were investigated to determine the predictors of adequate LM prescription (i.e. composite LM prescription score ≥18). The independent determinants of adequate LM prescription were: (a) number of patients seen per week (AOR: 0.98, 95% CI: 0.97 - 0.99, p-value= 0.006), (b) secondary care practice (AOR: 0.43, 95% CI: 0.20 - 0.90, p-value= 0.025) and (c) work experience (AOR: 1.05, 95% CI: 1.01 - 1.09, p-value= 0.01). However, when an interaction of total number of patients seen per week, secondary care practice and work experience were entered into a model, only the number of patients seen per week (seeing fewer patients) remained as the significant predictor of adequate LM prescription among these physicians ( AOR: 0.97, 95% CI: 0.95 - 0.99, p-value= 0.006).

**Table 1:** Prescription of lifestyle modification according to the physicians’ characteristics

Physicians’ characteristics	Lifestyle modification prescription				
	Salt restriction (n/%)	Weight control (n/%)	Tobacco cessation (n/%)	Healthy diet (n/%)	Physical exercise (n/%)
All (n=213)	191/89.7	201/94.4	202/94.8	173/81.2	160/75.1
**Sex**					
Female (n=81)	80/98.8	72/88.9	76/93.8	65/80.2	58/71.6
Male (n=132)	111/84.1	129/97.7	126/95.5	108/81.8	102/77.3
χ^2^/ p- value	11.7/0.001	7.4/0.007	0.27/0.60	0.08/0.78	0.86/0.35
**Experience**					
≤ 10 years (n=121)	114/94.2	113/93.4	111/91.7	90/74.4	82/67.8
>10 years (n=92)	77/83.7	88/95.7	91/98.9	83/90.2	78/84.8
χ^2^/ p- value	6.24/0.01	0.5/0.48	0.026*	8.59/0.003	8.09/0.004
**Level of practice**					
Primary (n=142)	122/85.9	133/93.7	131/92.2	125/88	117/82.4
Secondary (n=64)	62/96.9	62/96.9	64/100	41/64.1	42/65.6
Tertiary (n=7)	7/100	6/85.7	7/100	7/100	1/14.3
χ^2^ / p-value	6.56/0.04	1.88/0.39	5.8/0.06	18.3/<0.001	21/<0.001
**Type of practice**					
Private (n=155)	135/87.1	145/93.5	145/93.5	126/86.9	122/78.7
Public (n=58)	56/96.6	56/96.6	57/98.3	47/81	38/65.5
χ^2^ /p-value	0.044	0.52*	0.33*	0.0/0.97	3.93/0.047
**Knows that HTN onset can be prevented**					
Yes (n= 101)	93/92.1	98/97	101/100	88/87.1	87/86.1
No (n= 111)	97/87.4	102/91.9	100/90	84/75.6	72/64.9
χ^2^/p-value(Yes vs No)	1.25/0.26	2.69/0.26	10.56/0.001	4.53/0.03	12.76/<0.001
**Knows that HTN onset can be delayed**					
Yes (n=178)	157/88.2	167/93.8	167/93.8	139/78.1	132/74.2
No (n=35)	34/97.1	34/97.1	35/100	34/97.1	28/80
χ^2^/p-value(Yes vs No)	2.52/0.11	0.61/0.44	2.28/0.13	6.96/0.01	0.53/0.47

* Fisher’s exact. LM: Lifestyle modification, HTN: Hypertension

**Table 2:** Physicians’ prescription of lifestyle modification according to their practice type and patient load

LM prescription	Experience (years) (n)/ (x±SD)	p- value	Total no of patients (n)/ (x±SD)	p-value	No of HBP patients (n)/ (x±SD)	p-value
**Salt restriction**		**0.02**		**0.001**		**0.11**
Yes	(191)/ 11.74±9.75		(191)/ 69.08±27.54		(189)/20.63 ± 16.61	
No	(22)/ 16.23±7.58		(22)/ 89.86±16.36		(22)/ 14.73±11.75	
**Weight control**		**0.054**		**0.74**		**0.56**
Yes	(201)/ 12.52±9.74		(201)/ 71.07 ± 27.68		(199)/ 20.17± 16.14	
No	(12)/ 7.00±5.49		(12)/ 73.83±21.08		(12)/ 17.33±18.53	
**Tobacco cessation**		**0.01**		**0.07**		**0.51**
Yes	(202)/ 12.60±9.63		(202)/ 70.44±27.63		(200)/ 19.83±16.20	
No	(11)/ 5.00±6.63		(11)/ 85.82±15.15		(11)/ 23.18±17.65	
**Healthy diet**		**0.07**		**0.27**		**0.07**
Yes	(173)/ 12.79±9.83		(173)/ 70.23±28.83		(171)/ 19.03±15.87	
No	(40)/ 9.68±8.36		(40)/ 75.55±19.17		(40/ 24.18±17.38	
**Physical exercise**		**0.01**		**0.88**		**0.68**
Yes	(160)/ 13.21±9.63		(160)/ 71.39±28.66		(158)/ 20.27±16.89	
No	(53)/ 9.17±9.05		(53)/ 70.74±23.06		(53)/ 19.21±14.30	

LM: lifestyle modification, HBP: hypertensive

**Table 3:** The threshold for lifestyle modification prescription according to selected physicians’ characteristics

Physician’s Characteristics	Threshold for LM prescription		χ^2^ / p-value
	<140/90 mmHg (n/%) / (x±SD)	≥ 140/90 mmHg (n/%) / (x±SD)	
**No of physicians (n=213)**	11/5.2	202/94.8	
**Sex**			<0.001*
Female (n=81)	10/12.3	71/87.7	
Male (n=132)	11/0.8	131/99.2	
**Work experience (years)**	5.82 ± 7.71	12.55 ± 9.62	0.024
**Total patients seen/week**	54.55 ± 15.08	72.14 ± 27.57	0.037
**HBP patients seen/week**	6.27 ± 1.68	20.76 ± 16.35	0.004
**Type of practice**			0.03/0.86
Public (n=58)	5/8.6	53/91.4	
Private (n=155)	6/3.9	149/96.1	
**Level of practice**			9.97/0.007
Primary (n=142)	5/3.5	137/96.5	
Secondary (n= 64)	1/1.6	63/98.4	
Tertiary (n=7)	5/71.4	2/28.6	
**Knows that HTN onset can be prevented**			6.91/0.009
Yes (n=101)	1/1	100/99	
No (n=111)	10/9	101/91	
**Knows that HTN onset can be delayed**			7.12/0.008
Yes (n=178)	6/3.4	172/96.6	
No (n=35)	5/14.3	30/85.7	

* Fisher’s exact, LM: lifestyle modification, HTN: hypertension

## Discussion

The result of this survey suggests high level of prescription of all the five lifestyle interventions assessed in this cohort of GPs. Salt restriction was the most prescribed LM while physical exercise was the least prescribed LM. The high level of LM prescription for BP control is similar to that of Dutch and Cameroonian GPs but is in contrast to low level of LM prescription by German physicians [16-18]. These differences may be sequel to methodological differences. The use of open-ended questions in the German survey ensured spontaneous responses. On the other hand, the use of closed questions with pre-specified options from which the respondents chose their answers by the Dutch, Cameroon and current survey provided ample allowance for the overestimation of a desired practice such as LM prescription [16-18]. It is well-known that blacks generally have high salt sensitivity: which is usually heightened in hypertensive individuals [19,20]. The malleability of human taste for salt i.e. ability to decrease salt taste by gradual exposure to a lower dietary sodium is also well known [19,20]. Such knowledge by our GPs might explain the very high prescription of salt restriction. Regular physical exercise which was the least prescribed LM in our survey, had the highest prescription in the two European surveys mentioned earlier [16,17]. It is interesting to note that adherence to physical exercise was least in a study of LM adherence involving 404 hypertensive patients in Ethiopia, another African country [10]. The adherence in the Ethiopian study was 31.4%, 85.9%, 74.6%, and 69.1% to physical exercise, tobacco use cessation, moderation of alcohol intake, and dietary changes respectively [10]. The paucity of recreational facilities in Lagos, coupled with the hazards of walking/jogging on the streets of Lagos created by the absence of pedestrian's walkways on most roads and reckless riders of commercial motorcycles may underpin the GPs reluctance to prescribing physical exercise. Fear of adverse effects of physical exercise such as injuries especially in patients with co-morbidities also may have limited physical exercise prescription by these GPs.

Prehypertension is associated with cardiovascular risk that is intermediate between normotension and hypertension [21,22]. The term prehypertension was designated to identify individuals in whom early intervention by adoption of LM could decrease BP with reduction in the rate of progression to hypertension or outright prevention of its onset [22]. Hypertension guidelines recommend lifestyle interventions for individuals with prehypertension and high normal BP having one or more risk factors [5,22]. Uncertainty regarding when and how to implement LM by physicians has been documented as a barrier to effective BP control [14]. Majority (95%) of the respondents in this survey initiated LM prescription at BP ≥140/90mmHg. This not only suggests ignorance of the prehypertensive/high normal categorization of patients, but also the potential preventive benefits of LM prescription in this group of individuals. The above assertion is reinforced by the findings of the current study that physicians' knowledge of HTN prevention is associated with better LM prescription. A study by Isezuo et al. [23] reported a very high prevalence of prehypertension (58.7%) in Northern Nigeria. The high prevalence of prehypertension coupled with low physicians' prescription of LM in prehypertension is an indicator of the huge population being denied the potential benefits of LM such as the prevention of hypertension onset. This exposes the gap between LM need and prescription in Nigeria. Since prehypertension is mostly associated with one or more cardiovascular risk factors such as obesity, diabetes and dyslipidaemia, the above scenario might be contributory to the high and burgeoning prevalence of hypertension and cardiovascular diseases in Nigeria [21,24]. Unlike the lower tiers of healthcare facilities, teaching, and evaluation of clinical conditions is the norm in tertiary care centres. This may explain the higher frequency of initiation of LM at BP <140/90mmHg by respondents practicing in facilities affiliated to tertiary care centres. This reinforces the case for physicians' continuing education.

Paradoxically, the knowledge that the onset of HTN can be prevented or delayed was associated with LM prescription at BP ≥140/90mmHg. Inadequate consultation time and training of GPs to address LM effectively and efficiently might be contributory to this [15]. The emergence of total patient load as the only significant physicians' characteristic that predicted adequate lifestyle modification prescription is seminal. This therefore suggests that heavy patient load is antithetical to adequate LM prescription and consequently BP control. Heavy patient load is not unusual in general medical practice [25]. This is compounded by the currently low doctor to population ratio (a global driver of poor health outcomes) of 4 per 10,000 population in Nigeria [26]. Worsening of this low ratio is increasingly being driven by poor renumeration, poor working environment, and inadequate medical equipment and infrastructure [27]. Interventions to improve doctor to population ratio including measures aimed at retaining health personnel in Nigeria may translate to improved LM prescription and BP control in Nigeria. In addition to addressing the factors mentioned above, the destination countries for the emigrating physicians should be encouraged to implement the WHO Global Code of Practice on the International Recruitment of Health Personnel [28].

**Limitations of the study**: this study may be limited by its retrospective nature: which makes it prone to recall bias. The use of close ended self-reported questionnaire instead of an open ended one might heighten the potential of overestimating of LM prescription in this study. The survey addressed only Lagos based GPs, thus limiting its generalizability to the whole of Nigeria. However, Lagos has the highest concentration of healthcare workers in Nigeria.

## Conclusion

LM is widely prescribed for the treatment of hypertension but rarely prescribed for its prevention by general practitioners in Nigeria. Interventions to reduce physicians' patient load may engender improved LM prescription and HTN care by GPs in Nigeria. The burgeoning prevalence of hypertension and other CV diseases in Nigeria may be stemmed by adequate LM prescription for individuals whose blood pressure are in the prehypertensive or high normal range.

### What is known about this topic

- Lifestyle modification is a potent intervention and an essential strategy in blood pressure control;
- Implementation of lifestyle modification is more challenging than pharmacotherapy;
- Unhealthy lifestyle remains the most neglected aspect of hypertension control.

### What this study adds

- Lifestyle modification is widely prescribed for the treatment of hypertension but rarely prescribed for its prevention by general medical practitioners in Nigeria;
- Small patient load is a predictor of adequate lifestyle modification prescription by general medical practitioners in Nigeria.

## Competing interests

The authors declare no competing interests.
